# Diagnostic Performance of Self-Collected Saliva Versus Nasopharyngeal Swab for the Molecular Detection of SARS-CoV-2 in the Clinical Setting

**DOI:** 10.1128/Spectrum.00468-21

**Published:** 2021-11-03

**Authors:** Mohammad Khaja Mafij Uddin, Tahmina Shirin, Mohammad Enayet Hossain, Ahmed Nawsher Alam, Jenifar Quaiyum Ami, Rashedul Hasan, Mojnu Miah, Nusrat Jahan Shaly, Shahriar Ahmed, S. M. Mazidur Rahman, Mustafizur Rahman, Sayera Banu

**Affiliations:** a Infectious Diseases Division, icddr,b, Dhaka, Bangladesh; b Institute of Epidemiology Disease Control and Research (IEDCR), Mohakhali, Dhaka, Bangladesh; c Nutrition and Clinical Services Division, icddr,b, Dhaka, Bangladesh; Montefiore Medical Center and Albert Einstein College of Medicine

**Keywords:** SARS-CoV-2, COVID-19, RT-qPCR, *C_T_* value, saliva, nasopharyngeal swab

## Abstract

Coronavirus disease 19 (COVID-19)—caused by severe acute respiratory syndrome coronavirus 2 (SARS-CoV-2)—has spread rapidly around the world. The global shortage of equipment and health care professionals, diagnostic cost, and difficulty in collecting nasopharyngeal swabs (NPSs) necessitate the use of an alternative specimen type for SARS-CoV-2 diagnosis. In this study, we investigated the use of saliva as an alternative specimen type for SARS-CoV-2 detection. Participants presenting COVID-19 symptoms and their contacts were enrolled at the COVID-19 Screening Unit of Dhaka Hospital of the International Centre for Diarrhoeal Disease Research, Bangladesh (icddr,b), from July to November 2020. Paired NPS and saliva specimens were collected from each participant. Reverse transcription-quantitative PCR (RT-qPCR) was performed to detect SARS-CoV-2. Of the 596 suspected COVID-19-positive participants, 231 (38.7%) were detected as COVID-19 positive by RT-qPCR from at least 1 specimen type. Among the positive cases, 184 (79.6%) patients were identified to be positive for SARS-CoV-2 based on NPS and saliva samples, whereas 45 (19.65%) patients were positive for SARS-CoV-2 based on NPS samples but negative for SARS-CoV-2 based on the saliva samples. Two (0.5%) patients were positive for SARS-CoV-2 based on saliva samples but negative for SARS-CoV-2 based on NPS samples. The sensitivity and specificity of the saliva samples were 80.3% and 99.4%, respectively. SARS-CoV-2 detection was higher in saliva (85.1%) among the patients who visited the clinic after 1 to 5 days of symptom onset. A lower median cycle threshold (*C_T_*) value indicated a higher SARS-CoV-2 viral load in NPS than that in saliva for target genes among the positive specimens. The study findings suggest that saliva can be used accurately for diagnosis of SARS-CoV-2 early after symptom onset in clinical and community settings.

**IMPORTANCE** As the COVID-19 pandemic erupted, the WHO recommended the use of nasopharyngeal or throat swabs for the detection of SARS-CoV-2 etiology of COVID-19. The collection of NPS causes discomfort because of its invasive collection procedure. There are considerable risks to health care workers during the collection of these specimens. Therefore, an alternative, noninvasive, reliable, and self-collected specimen was explored in this study. This study investigated the feasibility and suitability of saliva versus NPS for the detection of SARS-CoV-2. Here, we showed that the sensitivity of saliva specimens was 80.35%, which meets the WHO criteria. Saliva is an easy-to-get, convenient, and low-cost specimen that yields better results if it is collected within the first 5 days of symptom onset. Our study findings suggest that saliva can be used in low-resource countries, community settings, and vulnerable groups, such as children and elderly people.

## INTRODUCTION

Coronavirus disease 19 (COVID-19), a novel coronavirus-induced pneumonia caused by severe acute respiratory syndrome coronavirus 2 (SARS-CoV-2), was first recognized and confirmed in December 2019 in Wuhan, China (1). Hence, it has spread exponentially to many countries, becoming a global pandemic, affecting 104,911,186 people worldwide, and triggering 2,278,579 deaths as of 4 February 2021, according to WHO records (2). Fast and precise diagnostic tests are mandatory for controlling the ongoing COVID-19 pandemic. The existing gold standard specimens include nasopharyngeal (NPS) or oropharyngeal (OPS) swab collection followed by reverse transcription-quantitative PCR (RT-qPCR) for the detection of SARS-CoV-2. A series of drawbacks raised during NPS or OPS collection paves the way for finding an alternative diagnostic specimen type (3). The collection of NPS involves inserting a swab stick into the rear side of the nasal cavities, which may induce discomfort and stimulate coughing and sneezing (4). Thus, medical staff may also be exposed accidentally during collection (5). NPS collection has been related to complex, unreliable, false-negative, and inconsistent test results, which may be due to the technical difficulties of appropriate swab collection (6). Moreover, NPS collection is not suitable for large-scale screening owing to the shortage of personnel protective equipment (PPE), viral transport media (VTM), and other logistics (7). Furthermore, the self-collection of NPS is difficult and less sensitive for virus detection (8).

Based on the above disadvantages of NPS, an alternative and safe specimen is needed urgently. Noninvasive and self-collected saliva could be an alternative to NPS for SARS-CoV-2 detection. There are several mechanisms underlying the existence of SARS-CoV-2 in saliva. In addition to other viruses, SARS-CoV-2 may migrate to the oral cavity from the upper/lower respiratory tract or may be released from the infected salivary glands (9). SARS-CoV-2 detection with saliva permits specimen collection at home, in outpatient clinics, or even in the community. The self-collection of saliva is painless, stress-free, easy to accept, and reduces hospital-acquired infections (10). Saliva can be used in comprehensive or epidemiological studies and is particularly useful for specific populations, such as children (11). Moreover, saliva collection alleviates the requirement of certified swabs, VTM, collection receptacles, and PPE, thus reducing diagnostic-related costs.

Since the COVID-19 outbreak, several studies have evaluated using saliva for the detection of SARS-CoV-2. In most of these studies, either the sample size was limited or they enrolled patients with confirmed COVID-19. Among the admitted patients and the early onset of disease, the viral load was higher in saliva samples than that in the routinely used NPS (12–14). In this cross-sectional study, we aimed to investigate the feasibility and utility of self-collected saliva for SARS-CoV-2 detection and compared the results with those of the health care-assisted NPS of ambulatory patients that were presented at the dedicated COVID-19 Screening Unit of Dhaka Hospital of the International Centre for Diarrhoeal Disease Research, Bangladesh (icddr,b).

## RESULTS

A total of 596 paired NPS and saliva samples were obtained from patients suspected to have COVID-19 who were either symptomatic or in contact with confirmed COVID-19 cases. Among them, 229 (38.4%) were detected as COVID-19 positive by RT-qPCR from the NPS. A total of 136 (59.3%) participants were male, and the median (interquartile range [IQR]) age was 35 (28 to 47) years among the confirmed COVID-19 cases. Most participants had mild-to-moderate symptoms. Mild cases were defined as individuals with COVID-19 symptoms without shortness of breath. Moderate cases were defined as individuals who had symptoms of pneumonia with shortness of breath.

During enrollment, the most common symptoms among the COVID-19-confirmed patients were fever (89.0%), cough (65.1%), loss of appetite (56.8%), altered smell (43.7%), runny nose (42.8%), chills (40.6%), and muscle aches (40.2%). Fifty (8.4%) of the participants were asymptomatic. Among the asymptomatic cases, 6 (12%) were positive for COVID-19 by NPS. The frequencies of fever, altered smell, and loss of appetite were significantly higher (*P < *0.001) among the COVID-19-confirmed cases than those of the participants who tested negative for COVID-19 (Table 1).

**TABLE 1:** Demographic and clinical characteristics of participants under investigation with RT-qPCR for COVID-19 diagnosis

Characteristic	Total patient data (*n* = 596)^*a*^	Patient data by COVID-19 infection status^*a*^		*P* value
		Infected (*n* = 229)	Uninfected (*n* = 367)	
Male	338 (56.7)	136 (59.3)	202 (55.0)	
Age (yrs) (median [IQR])	32 (26–42)	35 (28–47)	31 (26–39)	
Fever	473 (79.4)	204 (89.0)	269 (73.3)	<0.001
Sore throat	208 (34.9)	76 (33.2)	132 (35.9)	0.488
Chills	211 (35.4)	93 (40.6)	118 (32.1)	0.035
Runny nose	265 (44.5)	98 (42.8)	167 (45.5)	0.517
Cough	358 (60.0)	149 (65.1)	209 (66.9)	0.049
Shortness of breath	82 (13.7)	39 (17.0)	43 (11.7)	0.066
Altered smell	210 (35.2)	100 (43.7)	110 (30.0)	<0.001
Headache	224 (37.5)	91 (39.7)	133 (36.2)	0.391
Muscle aches	201 (33.7)	92 (40.2)	109 (29.7)	0.008
Joint aches	149 (25.0)	64 (27.9)	85 (23.2)	0.154
Loss of appetite	269 (45.1)	130 (56.8)	139 (37.9)	<0.001
Asymptomatic	50 (8.4)	6 (2.6)	44 (112)	<0.001

a All data are *n*(%) except where otherwise noted.

A total of 231 (38.75%) patients were confirmed to be COVID-19 positive by RT-qPCR from at least 1 specimen type. The positivity rates for COVID-19 from NPS and saliva specimens were 38.4% (229/596) and 30.9% (184/596), respectively. Among the 229 positive specimens, 184 (80.3%) were detected in both NPS and saliva, while 45 (19.6%) were positive for NPS but negative for saliva. There were 2 (0.5%) specimens that had viral RNA detection in saliva but were negative for NPS (Table 2). Among the six asymptomatic COVID-19-positive cases detected by NPS, two were detected as positive by saliva. To determine the test performance of the saliva, RT-qPCR results of NPS were used as a reference standard. The sensitivity and specificity of the saliva sample RT-qPCR were 80.3% (95% confidence interval [CI], 74.6% to 85.3%) and 99.4% (95% CI, 98.0% to 99.9%), respectively. The positive and negative predictive values were 98.9% (95% CI, 95.8% to 99.7%) and 89.0% (95% CI, 86.2% to 91.3%), respectively. From the analysis, the agreement between saliva and NPS specimens revealed 92% agreement (κ coefficient, 0.83; 95% CI, 0.78 to 0.87; *P* < 0.001).

**TABLE 2:** Detection of SARS-CoV-2 viral RNA in NPS and saliva specimens^*a*^

Saliva	NPS (*n*, %)			Agreement (%)	Kappa (κ) (95% CI)	*P* value
	Positive	Negative	Total			
Positive	184 (80.3)	2 (0.5)	186 (31.2)	92.11	0.83 (0.78–0.87)	<0.001
Negative	45 (19.7)	365 (99.5)	410 (68.8)			
Total	229 (100)	367 (100)	596 (100)			

a Total *n* = 596.

**FIG 1:**
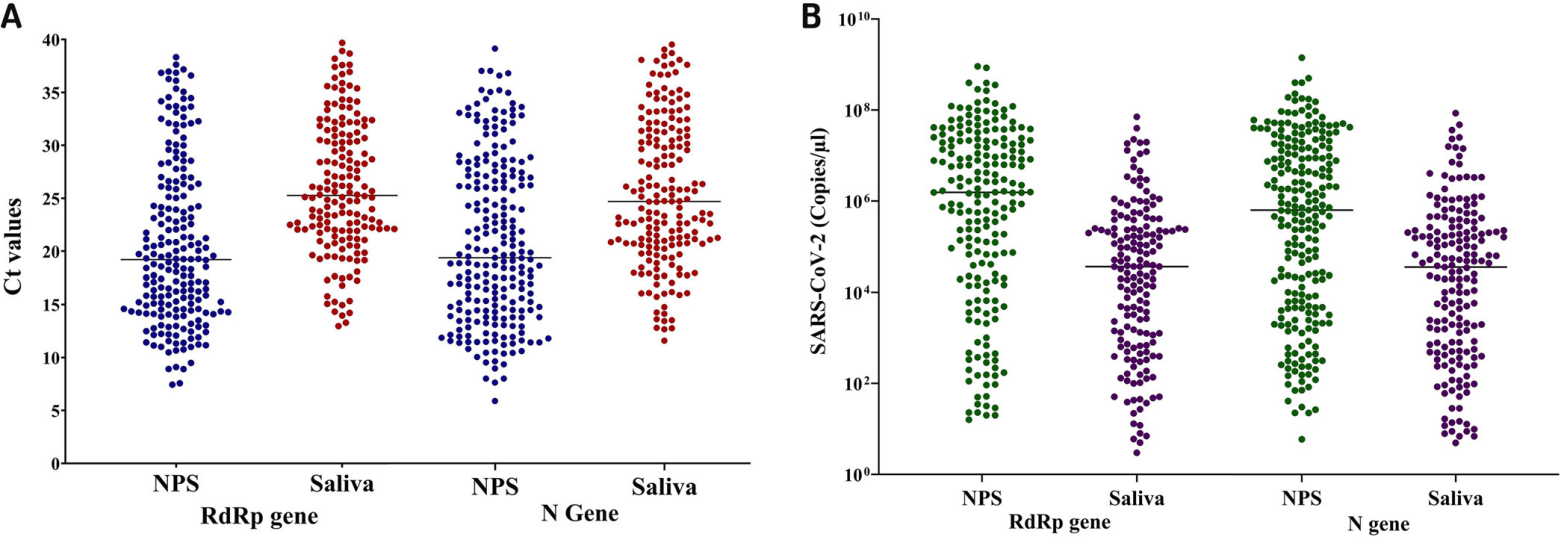
(A) Comparison of *C_T_* values of *RdRp* and *N* gene targets in paired NPS and saliva specimens. (B) Quantification of SARS-CoV-2 virus titer both in NPS and saliva specimens.

The median cycle threshold (*C_T_*) values were calculated for the *RdRp* and *N* genes in the SARS-CoV-2-detectable NPS and saliva samples. The median (IQR) *C_T_* values were 25.3 (22.0 to 31.1) and 25.1 (21.1 to 31.6) for *RdRp* and *N* gene targets, respectively, in saliva specimens, while the median (IQR) *C_T_* values were 17.7 (12.9 to 24.2) and 19.8 (15.0 to 27.4) for *RdRp* and *N* genes, respectively, in NPS (Fig. 1A). A total of 47 participants had discordant results, including 2 participants with virus detected in saliva but not in NPS and 45 participants with virus detected in NPS but not in saliva samples. The median *C_T_* value for these 45 discrepant participants was 29.2 (27.4 to 33.3). Among the 45 discrepant participants, 4 were asymptomatic, and the remaining 41 participants had nonspecific symptoms (e.g., runny nose, muscle aches, headache, and diarrhea) except fever. It was also noted that the virus titers (1.9 × 10^6^ and 3.4 × 10^2^) of two discrepant specimens (positive in saliva but negative in NPS) were lower than those found typically from other specimens.

We measured the SARS-CoV-2 viral RNA copy numbers in the NPS and saliva specimens. In this study, our assay detection limits were 16 and 14 copies/μl of NPS and saliva samples, respectively, at *C_T_* values of 37. A significantly higher number of SARS-CoV-2 RNA copies were found for both the *RdRp* and *N* genes in the NPS than that in the saliva. For the *RdRp* gene, the median log copies/μl were 1.5 × 10^6^ and 3.6 × 10^4^ in NPS and saliva samples, respectively. Similarly, for the *N* gene, the median log copies/μl were 6.8 × 10^5^ and 3.6 × 10^4^ in NPS and saliva samples, respectively (Fig. 1B).

**FIG 2:**
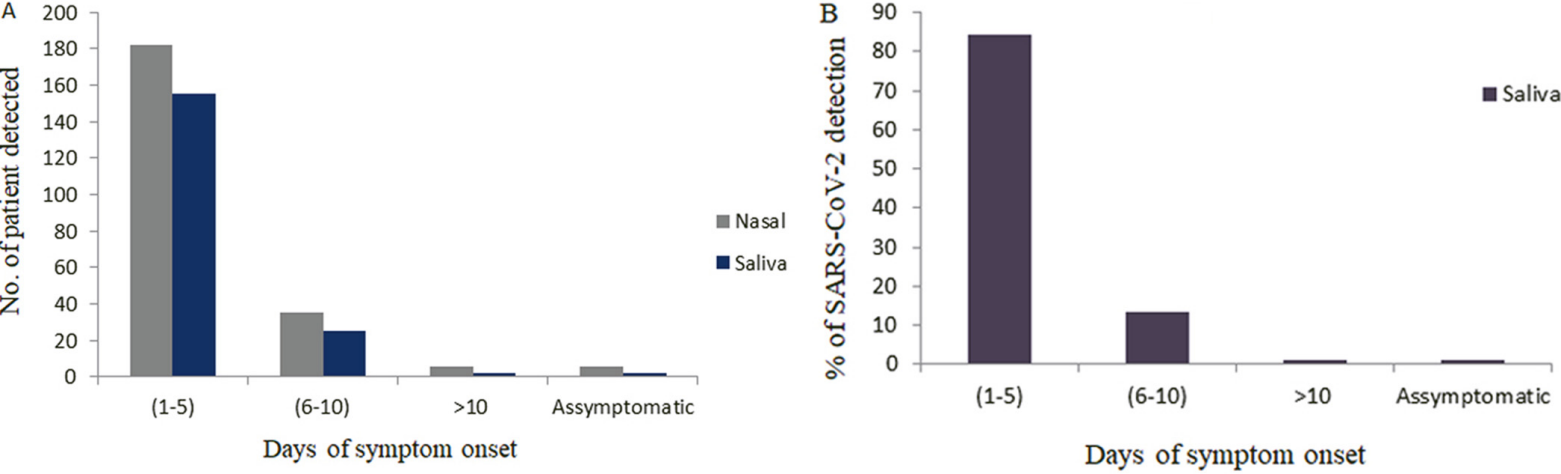
(A) Comparison of number of positive patients detected in NPS and saliva samples based on the days of symptom onset and asymptomatic cases. (B) Percentage of positivity of SARS-CoV-2 detection from saliva samples at 1 to 5 days, 6 to 10 days, and >10 days of onset of symptoms and asymptomatic cases.

We also calculated the number of positive patients and the percentages of positivity for SARS-CoV-2 in NPS and saliva samples in the early phase (1 to 5 days), progressive phase (6 to 10 days), and late phase (10 or more days) of symptom onset and in asymptomatic cases. A total of 182 and 155 patients were positive in NPS and saliva specimens, respectively, during 1 to 5 days of onset of symptoms (early stage), whereas the number of positive results decreased as the days of onset of symptoms increased (progressive and late stage of the disease) (Fig. 2A).

The sensitivity of saliva samples compared with that of NPS to detect SARS-CoV-2 from early phase, progressive phase, late phase, and asymptomatic patients was 85.2% (95% CI, 79.1% to 89.9%), 71.4%, 33.3%, and 33.3%, respectively. Among the saliva samples, SARS-CoV-2 detection was significantly higher (84.2%, *P < *0.001) among the patients who visited the clinic within 1 to 5 days of symptom onset. The detection rate was 13.6% for 6 to 10 days and 1.1% for more than 10 days of onset of symptoms and asymptomatic patients, respectively (Fig. 2B).

## DISCUSSION

Fast and precise diagnosis is one of the key issues regarding epidemic preventive measures for any contagious disease, especially in the absence of effective therapeutic agents or vaccines. The current study investigated the accuracy of using saliva specimens for the diagnosis of COVID-19, which has already taken the lives of more than 2.6 million individuals globally (15). The use of saliva has several advantages over NPS in the diagnosis of SARS-CoV-2. First, collecting saliva is a noninvasive procedure that avoids discomfort to the patients. Second, saliva can be collected at home or outside the hospital without the help of health care personnel. To date, studies evaluating saliva as an alternative specimen type for SARS-CoV-2 detection have been conducted largely among hospitalized confirmed COVID-19 patients (3, 8, 16, 17). It is crucial to understand the feasibility and suitability of the use of saliva for COVID-19 diagnosis in community-based screening facilities.

The present study was carried out in a community-based outpatient facility, where most of the suspected patients had mild to moderate or no symptoms. The RT-qPCR results of the saliva samples showed a sensitivity of 80.3% compared with the NPS. Earlier studies reported the sensitivity of RT-qPCR results of saliva samples of roughly from 83% to 86%, which is very similar to our findings. All of these studies were conducted with a limited number of samples (4, 18–20). Two other investigations with a limited sample size found the highest sensitivity with saliva samples (100%), and in both cases, only hospitalized COVID-19 patients were enrolled (13, 21). Another study reported 83.4% sensitivity, in which all suspected participants were hospitalized with acute clinical symptoms (22). The differences in the sensitivities among studies may be due to disease severity, the procedure of saliva collection and RNA extraction, and target genes used for amplification.

In the current study, the percentages of COVID-19 positivity for saliva and NPS were 84.2% and 79.5%, respectively, among the samples collected at the early phase of symptom onset (1 to 5 days). This result suggests that both specimens had similar sensitivities, at least at the early stage of infection. A previous study conducted by Ikeda et al. reported a 65.6% to 93.4% detection rate at the early stage of infection (3). Other studies have found similar results where sensitivities reflect the timing of symptom onset (3, 23). A lower positivity rate (55% to 63%) was also found among the samples collected at the early stage of symptom onset (24). In contrast, the sensitivity decreased gradually after 5 days of symptom onset (71.4% to 33.3%). A study conducted by Becker et al. reported the lowest sensitivity (69.2%) with saliva samples collected at the late stage of the onset of symptoms (25). Our study findings would help policymakers to consider using saliva samples for SARS-CoV-2 detection at the early stage of symptom onset in the case of mass detection.

In our study, the median *C_T_* values were lower for NPS, indicating a higher viral load for NPS than that for saliva for both *RdRp* and *N* genes. Several studies have also reported a lower level of viral load in the saliva than that in NPS or throat swabs (17, 20, 26). Wyllie et al. reported a higher viral load in saliva during the first week of symptom onset and it declined gradually after the onset of symptoms (8). Higher SARS-CoV-2 viral RNA was also detected in the posterior oropharyngeal saliva samples at the time of disease presentation (14, 27). It is well established that viral load is associated with disease severity. Enrollment of hospitalized confirmed COVID-19 cases, collection instructions (avoiding food, water, and brushing before sample collection), and use of early-morning saliva may be the possible reasons for higher viral load in saliva among different studies. In our study, the viral load was lower in the saliva than that in the NPS. This result might be owing to the saliva collection procedure, patient enrollment, and target genes used for amplification. The addition of VTM, liquid Amies medium, phosphate-buffered saline (PBS), or any other maintenance medium can prevent enzymatic degradation and homogenize the saliva samples. In this study, saliva samples were collected by walking without prior instructions in a sterile container without any transport medium. The collection procedures and transportation factors may have an impact on the assay detection accuracy. In addition, saliva mixed with sputum may hamper RNA extraction and PCR amplification.

Among the 45 discrepant specimens, the median *C_T_* value (29.2; IQR, 27.4 to 33.3) was higher among the NPS than the median *C_T_* value of common positive (25.3; IQR, 22.0 to 31.1) samples, indicating that the viral load was beyond the detection level in these saliva specimens. For two other discrepant specimens that were positive in saliva but negative in NPS, repeated RT-qPCRs with the same specimens showed similar results. This result may have occurred because of the inappropriate NPS collection or the quality of the collected specimen (20).

Our study had several strengths. We enrolled participants, who were at a high risk of occurrence of COVID-19, with contacts of confirmed COVID-19 cases that were either symptomatic or asymptomatic. We collected paired NPS and saliva specimens at the same time from each participant, which may reduce the chance of variation in viral load among the specimens. One of the limitations of the study was that some of the self-collected saliva samples were mixed with sputum or mucus, which inhibited the RNA extraction as well as RT-qPCR analysis.

In conclusion, the study findings provide more evidence to suggest that saliva can be used as an alternative specimen type for SARS-CoV-2 detection. The sensitivity of saliva specimens increased up to 85.2% at the early stage of infection, indicating that it can be used accurately for the diagnosis of COVID-19 early after symptom onset. Although the sensitivity of saliva is lower than that of NPS, it can be used for COVID-19 diagnosis in several settings, including high-incidence, low-resource countries; community settings; and vulnerable groups, such as children or elderly people.

## MATERIALS AND METHODS

### Study setting.

This study was conducted at the COVID-19 Screening Unit of the Dhaka Hospital (icddr,b). This designated COVID-19 screening unit was accessible only to staff members and their dependents. During the study period, on a daily average basis, 35 to 40 suspected patients and contacts of confirmed COVID-19 cases with or without the signs and symptoms of COVID-19 visited this screening unit. Suspected participants aged more than 10 years were eligible, and informed written consent was obtained from each participant before enrollment. Biological specimens and demographic and clinical data, including signs and symptoms of COVID-19, history of comorbidities, hospitalization, and traveling history, were collected from all participants. The study was approved by the Research Review Committee and the Ethical Review Committee of icddr,b.

### Sample collection.

A total of 596 paired NPS and saliva samples were collected from the participants. The NPS was collected in the VTM by a trained nurse according to the standard procedure described previously (28). Healthcare workers instructed the participants to avoid taking food, drinking water, brushing teeth, washing mouth, coughing, and sniffing before saliva collection. Participants were asked to clean their hands with an alcohol-based hand sanitizer or soap and water prior to saliva collection. A flocked swab was kept under the tongue for 2 to 3 min to pool the saliva in the mouth and gently spit 1 to 2 ml of saliva into a sterile container without the transport medium. The saliva collection procedure was supervised by trained health care workers to ensure good quality and an appropriate volume of saliva from each participant. Paired saliva and NPS were stored in a cool box until transported to the laboratory (20).

### Viral RNA extraction and identification.

All NPS and saliva samples were processed for SARS-CoV-2 detection at the Virology Laboratory of the Institute of Epidemiology Disease Control and Research (IEDCR) and icddr. Viral RNA was extracted from both NPS and saliva samples (140 μl each) using the QIAamp viral RNA mini kit (Qiagen, Germany). RT-qPCR was performed using previously described primers and probes targeting the *RdRp* and *N* genes (29). The reaction system and amplification conditions were performed using the iTaq universal probes one-step RT-qPCR kit following the manufacturer’s instructions. RT-qPCR was carried out in a final reaction volume of 25 μl containing 12.5 μl of 2× PCR buffer, 0.5 μl of 50× RT enzyme, 1.0 μl of each primer (10 μM) and 0.5 μl of each probe (10 μM), and 5 μl of template RNA. The assay was performed on a CFX96 touch real-time PCR system (Bio-Rad Laboratories Inc. USA). The results were categorized as positive for SARS-CoV-2 when the *C_T_* values of one or both target genes were ≤37 whereas, the *C_T_* values of >37 were considered negative.

For viral load quantification, we designed a synthetic plasmid as a positive control, which was synthesized by GenScript (Hong Kong). We reconstituted 4 μg of dried control in Tris-EDTA (TE) buffer to make a stock with a copy number of 1.07 × 10^10^ copies/μl. The stock plasmid was 10-fold serially diluted several times ranging from 1.07 × 10^7^ to 1.07 × 10^3^ copies/μl to generate a standard curve. The viral RNA copy number was quantified per microliter of sample.

### Statistical analysis.

All collected data were entered and analyzed using the Statistical Package for the Social Sciences software (SPSS) version 20.0. Additional statistical analyses were performed using GraphPad Prism 9.0. Descriptive statistics for categorical variables are expressed as number percentages, and continuous variables are presented as median (with interquartile range [IQR]). Comparisons between two groups were performed using the Wilcoxon signed-rank test. Sensitivity, specificity, positive predictive value, and negative predictive values were determined to assess the diagnostic performance of the saliva specimens. A two-sided *P* value of <0.05 was considered statistically significant.
